# Near real-time change detection tool for photogrammetric flood preparedness

**DOI:** 10.1007/s10661-024-13597-9

**Published:** 2025-01-04

**Authors:** Michael Kögel, Dirk Carstensen

**Affiliations:** Technische Hochschule Nürnberg Georg Simon Ohm, Institute of Hydraulic Engineering and Water Resources Management, Nuremberg, Germany

**Keywords:** Change detection, Near real-time, Photogrammetry, Flood event, 2d-hn, M3C2

## Abstract

Through the mobilization of movable objects due to the extreme hydraulic conditions during a flood event, blockages, damage to infrastructure, and endangerment of human lives can occur. To identify potential hazards from aerial imagery and take appropriate precautions, a change detection tool (CDT) was developed and tested using a study area along the Aisch River in Germany. The focus of the CDT development was on near real-time analysis of point cloud data generated by structure from motion from aerial images of temporally separated surveys, enabling rapid and targeted implementation of measures. The differences identified in the study area using distance comparison (M3C2) were segmented into individual components and categorized. Subsequently, the data was compared to existing two-dimensional hydrodynamic numerical calculation results (HQ_100_). The implementation of the CDT is feasible for a variety of RGB camera-equipped aerial vehicles due to the point cloud-based analysis and postprocessing. By overlaying and visualizing the detected changes with numerical simulation results, a quick assessment of the hazard potential in the event of a possible flood can be made. In the case of the study area along the Aisch River, the localization of construction materials, a steel container with debris pile, and a motor vehicle in the flood hazard zone of a potential HQ_100_ event could be confirmed, although no mobilization of the materials was to be expected due to the expected hydraulic conditions of a flood event.

## Introduction

In recent years, the frequency and intensity of flood events in Germany and the European region have intensified. An analysis of temperature and precipitation trends by the German Environment Agency (In German: *Umweltbundesamt*) revealed an increase in precipitation especially during winter season (Umweltbundesamt, 2019).

The increasing imperviousness of land surfaces, along with human interventions in river morphology (such as the straightening of river systems and narrowing of flow cross-sections), results in the acceleration of flood waves and an intensification of peak discharge (Patt & Gonsowski, 2011). The *Guidelines for Forward-Looking Flood Protection* (in German: *Leitlinien für einen zukunftsweisenden Hochwasserschutz*) of the Federal-State Working Group on Water (LAWA, 1995) address the necessity of relocating dikes, unsealing areas, practicing site-appropriate agriculture and forestry, and restoring watercourses to preserve natural environmental resources. It is not possible to completely prevent a major flood event with certainty, but it is possible to limit the resulting flood damage (LAWA, 1995). Flood events must once again be understood as natural events to which humans are always exposed (EG, 2007; Patt & Gonsowski, 2011).

Based on the recommendations for the preparation of flood hazard and flood risk maps (in German: *Empfehlung zur Aufstellung von Hochwassergefahrenkarten (HWGK) und Hochwasserrisikokarten (HWRK)*, LAWA, 2010) and their updates in LAWA (2018), specific analytical tools have been developed that visualize the extent of significant flood events based on hydraulic parameters (typically flow velocity, water depth, and flow vectors) as well as a risk assessment for infrastructure.

The foundation of these maps primarily relies on the results obtained from two-dimensional hydrodynamic numerical (2d-hn) model simulations.

Risk assessment is typically conducted by comparing the probability of a flood event with the potential adverse consequences associated with flooding (flood damages) (Müller, 2010). The risk value is derived from the product of these two factors. The latter is also referred to as vulnerability, which serves as a measure of exposure, susceptibility, and the potential for damage in a flood event (Müller, 2010). Typically, this assessment is based on fixed and immovable infrastructure. The presence of movable materials, which can cause blockages (Heimerl, 2018) and damage to infrastructure and pose a threat to human lives, is not considered in these evaluations. Identifying movable objects can provide valuable insights into potential damage risks in the event of a flood, which can be mitigated through preventive measures.

Thus, the detection of potential hazards in the form of materials mobilized by a flood event is just as important as the high-frequency and near real-time monitoring of floodplain development. Through modern and alternative measurement techniques, it is possible to observe and record these processes relatively inexpensively, with high accuracy, minimal time expenditure and in near real-time.

### Remote sensing methods

A variety of surveying methods are available in the context of remote sensing of flood areas and identification of movable objects through the comparison of spatio-temporal information (change detection, CD). Due to rapid developments in unmanned aerial vehicles (UAVs) and the associated capabilities for employing airborne laser scanning (light detection and ranging, LiDAR, Greenwood et al. (2019)) and photogrammetry (particularly using structure from motion, SfM, Mancini and Salvini (2020)), study areas can be accurately surveyed at short intervals (Andresen & Schultz-Fellenz, 2023). SfM-based imaging represents a cost-effective method (Stead et al., 2019) as it can utilize simple RGB camera systems. The compact design of this combination enables extended flight times, even in close proximity to infrastructure. Moreover, surveys can be combined with multispectral or thermal cameras alongside RGB camera systems (Shi et al., 2024), thereby enhancing categorization possibilities.

Airborne laser scanning (ALS) is comparatively expensive and is often subject to restrictions regarding distances from infrastructure and human populations due to its larger mass. Many LiDAR systems offer the capability to analyze emitted and reflected laser signals across the entire laser spectrum (multi-echo, full waveform, Eltner et al. (2022)). Compared to single pulse data, the various (time-delayed) amplitudes of the reflected signals can be evaluated and attributed to corresponding objects (e.g., forest canopy, building roofs, and terrain surface) (Salas, 2021; Eltner et al., 2022). This makes LiDAR imaging frequently used for generating digital elevation models (DEM), as they allow extraction of ground information from the study area (Okyay et al., 2019).

In addition to airborne photogrammetric remote sensing, close-range photogrammetry is also commonly employed in monitoring applications, where data is gathered at predetermined intervals using fixed installations (such as camera systems). Blanch et al. (2021, 2023) describe a method for detecting pre-failure deformation and identifying rockfalls at high spatio-temporal resolution. An inherent limitation of this system, particularly in expansive study areas, is its narrow focus on specific observation zones, constraining the flexibility of data analysis.

### Analyzing change detection and flood areas

CD and the derivation of water surfaces can be performed in various ways, depending on the aforementioned recording methods. The analysis is typically based on 3d datasets (point clouds, PC) or 2d and 2.5d datasets (raster data). According to Qin et al. (2016), 2d change detection offers several advantages over 3d change detection, particularly in its suitability for investigating individual buildings. It is easy to implement, and the data collection process is straightforward. However, this approach is significantly affected by altitude and atmospheric conditions, and it is limited by viewing angles, which can lead to perspective distortions.

On the other hand, as noted by Qin et al. (2016), 3d change detection is advantageous for covering robust terrains and provides height information from oblique views, effectively minimizing perspective effects, especially in very high-resolution data. Nonetheless, this method can yield unreliable 3d information that may result in artifacts, and the data sources can still be relatively expensive.

Spatio-temporal differences can be identified and quantified through distance measurements using 3d geometric attributes (e.g., from PC generated by SfM and LiDAR and using the Multiscale Model-to-Model Cloud Comparison (M3C2) approach as presented in Lague et al. (2013)).

Due to the absence of height information in 2d datasets, analysis in raster data is generally performed by comparing adjacent pixels based on predefined features (semantic segmentation). Alternatively, if height information (2.5d) is available, datasets are compared geometrically (DEM of differences (DoD), Bailey et al. (2022)), often involving significant height averaging.

The possibilities and methods of analysis processes have greatly expanded in recent years due to the developments in AI (Long et al., 2014; Heipke & Rottensteiner, 2020; Panahi et al., 2021). Machine/deep learning algorithms can be trained supervised and unsupervised. Common supervised learning methods include support vector machines, where a hyperplane is defined to maximize the margin distance from nearby points for separating different classes (Eltner et al., 2022), and random forest analyses, where random forests serve as ensemble classifiers consisting of individual classification and regression trees (Breiman, 2001).

Convolutional neural networks (CNN) have gained significant importance due to their versatility and capability, frequently used as tools for image and raster analysis, including flood mapping (Gebrehiwot et al., 2019) and change detection (Farmakis et al., 2022).

K-means cluster analysis is an unsupervised learning tool frequently integrated into various software applications, offering extensive possibilities for segmenting data by clustering adjacent pixels, such as in urban flooding risk assessments. (Xu et al., 2018).

Open access to satellite imagery and the ability to access and analyze remote sensing datasets almost globally have expanded raster analysis methods (Colacicco et al., 2024). This provides a large volume of data to train and optimize algorithms, offering significant potential to detect flood events (FE) and implement measures related to flood management (Kuntla, 2021).

Compared to ALS and SfM, the resolution of current satellite raster data, which usually ranges from 30 to 1 m (Sadiq et al., 2022), is relatively coarse, and data collection cannot occur as frequently, with time intervals ranging from hours to days. Particularly during FE, a quick response time is necessary, and data must be available with high accuracy and frequency. However, satellite imagery allows for the derivation of long-term time series, enabling the investigation of past events and the improvement of existing models and approaches (Sadiq et al., 2022; Schlaffer et al., 2022).

Synthetic aperture radar (SAR) enables data collection during cloud cover and at night, whereas satellites with optical imaging technology produce no or erroneous datasets during these periods (Bioresita et al., 2018; Tarpanelli et al., 2022). SAR technology takes advantage of the fact that the return signal of water covered areas is weak due to the signal getting reflected away (Tarpanelli et al., 2022; Xu, 2006; Xu et al., 2018). This occurs especially over static water surfaces without significant waveforms. Additionally, studies have already been conducted in relation to the identification of ice surfaces using both technologies, where the backscatter of the SAR signals depends on several factors, such as the surface structure of the ice, the thickness of the ice sheet, and the moisture in the ice sheet or the overlaying snow cover (Möldner et al., 2024).

Based on the aforementioned factors, ALS or SfM conducted via UAVs emerges as a suitable approach for performing high-precision imaging within an appropriate timeframe in the context of flood management. The 3d datasets generated by these methods offer insights about potential changes in the study area before a FE and facilitate real-time mapping of flooded areas during a FE. Additionally, the SfM method has the advantage of creating photorealistic images, thereby offering further visual analysis possibilities.

In this study, the change detection process was developed based on the SfM method using UAVs, due to the fact that it can be used with a variety of different measurement systems and in different study areas.

Extensive investigations have already been conducted in this area using aerial photogrammetry in conjunction with UAVs (Dinkel et al., 2020; Gebrehiwot & Hashemi-Beni, 2020; Mancini & Salvini, 2020; Li et al., 2021; Eltner et al., 2022; Andresen & Schultz-Fellenz, 2023).

### Near real-time analysis

Real-time analysis, where results are available at the exact moment of data acquisition, is not feasible due to the need for the deployment process to be completed first (Wienhold et al., 2023). Near real-time analysis, however, depends on the specific context and requirements of the investigation and cannot be defined by a fixed time frame, especially in remote sensing applications. On the one hand, fixed time-lapse monitoring systems and close-range photogrammetry (as shown in Blanch et al. (2020, 2023)) can capture changes within seconds and analyze them with short post-processing times, but they are limited to a relatively small area. On the other hand, satellite images cover very large areas at intervals of several days (Colacicco et al., 2024) but can be processed in near real-time in less than 1 min (Katiyar et al., 2021).

The aim of this study was to reduce and optimize the post-processing duration sufficiently to classify the total duration of the change detection tool as near real-time compared to alternative measurement methods. Preparatory work for this had already been conducted in Kögel and Carstensen (2024) by optimizing and reducing the survey duration.

## Study area

**Fig. 1:**
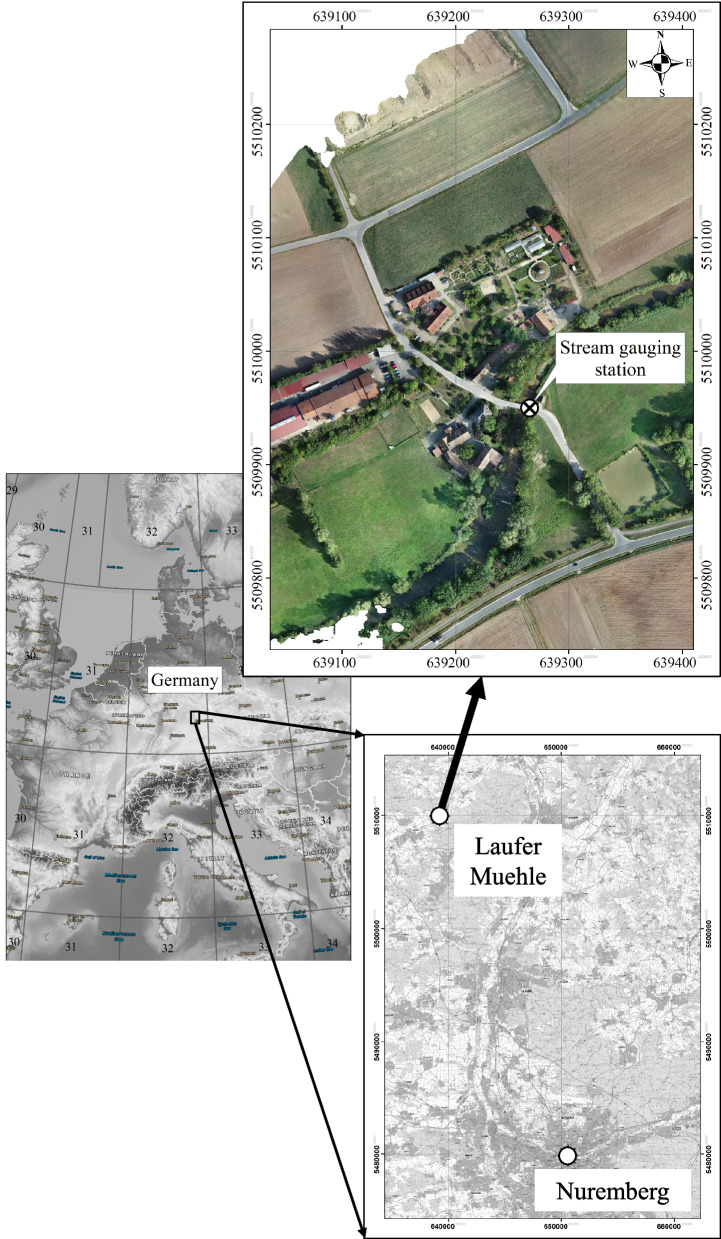
Location of the Laufer Muehle area and the stream gauging station (map on the left: OpenStreetMap contributors, (n.d.); map lower right corner: Bayerische Vermessungsverwaltung, (n.d.))

As described in Kögel and Carstensen (2024), the study area (Laufer Muehle) is an approximately 0.4 km^2^ section along the Aisch River in Bavaria near Nuremberg. The area is influenced by anthropogenic factors and a stream gauging station is located there. Further details about the study area (e.g., main values of water level and discharge) are provided in Kögel and Carstensen (2024). Figure 1 illustrates the location of the study area.

## Methods

### Developing a cost-efficient, straightforward change detection tool

A change detection tool (CDT) was developed to automate the analysis of multitemporal changes based on aerial photogrammetry. This tool uses distance comparisons between PCs and multitemporal aerial imagery to reveal differences in the study area. This makes it possible to identify potential movable objects in the study area that could pose a high hazard potential during an FE.

The focus in developing the CDT was on enabling identification while considering the following aspects:

- Relatively low preparation effort (e.g., without the need for pre-trained classifiers)
- Compatibility with a wide range of UAVs with RGB camera systems
- No requirement for additional measuring equipment or reference objects (such as ground control points (GCP))
- Use of conventional RGB aerial imagery
- Applicability in diverse environments
- Independence from seasonal changes (by filtering out vegetation-related differences)
- A user-friendly format (compact presentation of results and easy estimation of required actions)

As discussed in the “Analyzing change detection and flood areas” section and evident from the preceding points, the decision was made to use the combination of SfM and UAV-based imaging for implementing the CDT.

In the investigations by Kögel and Carstensen (2024), a feasibility study was conducted to examine which recording parameters of a DJI Phantom 4 RTK UAV unit can efficiently represent changes in a study area based on multitemporal surveys. The approach was chosen to balance minimal survey time with the required accuracy of the PCs generated by SfM. As a result, a configuration of survey parameters was determined, allowing a 0.4 km^2^ area to be captured in 15.5 min. In this context, initial processing identified and marked movable objects in the study area. The detection of multitemporal differences was performed through distance comparison using the Multiscale Model-to-Model Cloud Comparison (M3C2) method described in Lague et al. (2013). Compared to the cloud-to-cloud distance determination, such as that conducted in Kögel et al. (2022), this method has the advantage that existing normal values of individual measurement points could be applied for distance determination without needing to be calculated first. Since these typically arise with good accuracy during the SfM process, additional postprocessing time could be avoided.

In the investigations by Kögel and Carstensen (2024), the postprocessing of aerial images using the SfM method was based on parameters derived from experience and proven effective in previous studies. However, this did not rule out the possibility that better postprocessing parameters might exist, which could lead to comparable results in a shorter processing time due to increased efficiency in the interaction between calculation duration and accuracy.

Based on previous investigations, further insights into the influences of different postprocessing parameter configurations were necessary to minimize the time required for the overall processing for near real-time analysis of CD.

The aim of this study was to identify a combination of processing parameters along with optimized acquisition parameters that minimizes the total duration of the identification process while maintaining acceptable accuracy of results. The observed multitemporal changes in the study area were divided into individual components to characterize specific objects. An estimation of potential risks of mobilization during a FE could be made by integrating the detected changes with existing two-dimensional hydrodynamic-numerical (2d-hn) simulation results. The CDT results were compiled in a report (PDF-document).

Since the CDT relies on a variety of software applications, programming languages, and interfaces, the processes were transferred to a GUI through which all parameters can be defined and initialized. This makes using the tool easier and more robust to false settings (Fig. 4).

### Measuring device

For aerial imaging, a DJI Phantom 4 RTK was employed using Real-Time Kinematic (RTK), and the Satellite Positioning Service of the German National Survey (SAPOS). This setup enables horizontal position accuracy within the range of 1–2 cm and vertical position accuracy within 2–3 cm (SAPOS, 2015).

### Computer hardware: high performance cluster

The main part of the computations was carried out on a high performance cluster (HPC). The HPC comprises a total of six nodes, each equipped with 56 CPU dedicated to computations. Additionally, one of the nodes is equipped with a PNY Quadro RTX 4000 GPU, utilized for SfM calculations.

The capability to selectively target nodes and CPUs for computations enabled the execution of parallel and sequential calculation processes. The SfM calculations were notably accelerated through the utilization of a GPU. Point cloud computations primarily utilize CPU and RAM capacities. Consequently, more computational resources (five nodes) were allocated for these calculations compared to the SfM calculations, which were limited to the node equipped with the GPU.

### Flight and data acquisition parameters

The selected flight parameters were derived from the investigation results presented in Kögel and Carstensen (2024), leading to good accuracy results with the utilized measurement system. Accuracy was validated using a dataset generated through the implementation of georeferenced (RTK) GCPs in the SfM process. The utilized acquisition parameters are listed in Table 1.

**Table 1:** Optimized flight parameters for surveying in the Laufer Muehle area using a DJI Phantom 4 RTK (Kögel & Carstensen, 2024)

Flight parameter	Value
Flight mode (normal grid/double grid) [-]	Normal grid
Flight altitude [m]	110
Ground sampling distance (GSD) [cm]	3.01
Flight velocity [m/s]	4
Camera tilt[°]	60
Horizontal overlap [%]	60
Vertical overlap [%]	60
Shutter priority [-]	No

Surveying at Laufer Muehle was conducted in September 2022. The acquisition was performed twice with the same survey parameters. In this study, suitable processing parameters for change detection were identified by comparing these two datasets, aiming for a relatively low post-processing duration and minimal error (root mean square (RMS) of the M3C2 distances), ideally close to 0 m. After determining suitable postprocessing parameters through, these parameters were applied to the comparison of multitemporal acquisitions.

### Data processing for analyzing change detection

#### SfM

Agisoft Metashape 1.8.4 was used for the photogrammetric reconstruction of aerial images using SfM. The software provides a solid foundation for the automated processes of the CDT, particularly due to the ability to perform automated workflows using Python.

Using SfM, a point cloud reference dataset (Ref) was created based on the photographs taken during survey 1. A second point cloud was generated as the compare dataset (Com) through SfM and images captured at a later time (survey 2). These processes resulted in the point clouds that were compared to analyze CD.

#### Point cloud postprocessing

A portion of the processing and computation steps was performed using the open-source software CloudCompare (CC) 2.12.4. Initially, the point clouds generated by SfM were filtered based on their confidence values. This value is derived from the SfM process and corresponds to the number of depth maps used to generate a point and shows how reliably a point represents reality. The confidence values can range between 1 and 255, with 1 indicating very low confidence and vice versa (Agisoft LLC, 2022a). As a result, erroneous or inaccurate points (e.g., due to vegetation movement and noise) were removed. The investigations showed that a confidence value of 5 was effective in filtering most inaccurate points without excessively thinning the remaining PCs.

The distance comparison between the reference and compare PCs was conducted using the M3C2 distance comparison method as described in Lague et al. (2013). An appropriate parameter combination for the M3C2 calculation for this investigation area had been previously determined through a sensitivity analysis in Kögel and Carstensen (2024) and is shown in Table 2.

**Table 2:** Optimized M3C2 parameters developed from a sensitivity analysis (Kögel & Carstensen, 2024)

Projection scale [m]	0.8783
Max depth [m]	2.5
Normal computation [-]	Normals from SfM process

Changes in the study area were determined based on the calculated M3C2 distances. The M3C2 distance difference in areas with little or no change would be a at least few centimeters (due to unequal point distribution and positional inaccuracies) with ideal alignment and accuracy of the PCs calculated by SfM. However, discrepancies arising from SfM calculations and inaccurate positioning of the images can generally result in noticeable distance differences even in stable areas. Such inaccuracies can often be minimized through co-registration, where two PCs representing the same area are aligned. Nota et al. (2022) demonstrated that co-alignment of a dataset from surveying using general GNSS with at least one RTK survey improved the accuracy to the level of an RTK survey. However, Lague et al. (2013) obtained poorer results through co-registration, as the vegetation was automatically removed prior to point-cloud comparison, and Eltner et al. (2022) describe the problem that, during cloud-to-cloud registration, areas of change might be confused with stable areas. Therefore, it is essential to conduct optimization measures based on the specific study area. Given that these investigations focused on the adaptability to a wide range of study areas, optimization through co-registration was disregarded.

The dataset was filtered based on the M3C2 distance to neutralize minor changes that could have marginal negative impacts during a flood event, as well as measurement points resulting from noise effects in the calculation results. Filtering distances has proven to be an effective technique in this context.

Using the Label Connected Components (LCC) (Girardeau-Montaut (2015a)) algorithm implemented in CC, it is possible to divide the PC into individual components based on the parameters minimum points per component (*n*_pts,min_) and a minimum distance between components (octree level, OL). Both parameters strongly depend on the properties of the input PC and cannot be universally determined by a single parameter combination. The corresponding values must be determined in parallel computational steps to enable automated categorization using LCC.

The octree structure is used in many spatial computation procedures in CC. According to Girardeau-Montaut (2015b), PCs in CC are represented within a cubic volume (bounding box). By default, the dimensions of the bounding box are derived from the maximum *x*, *y*, and *z* distances. The maximum distance of one of the three coordinates corresponds to the bounding box dimensions in all three spatial directions.

The bounding box is subdivided into eight equivalent sub-cubes per octree level. The resulting cell size of the sub-cubes, and consequently the octree distance, can be calculated using Eq. (1). Therefore, defining a high OL results in a small distance between the measurement points.

1 $$\mathrm{cell}\;\mathrm{size}(\mathrm{OL})=\frac{{\mathrm{d}}_{{\mathrm{max}}_{\mathrm{x},\mathrm{y},\mathrm{z}}}}{2^\mathrm{OL}}$$

OL: octree level
$${\mathrm{d}}_{{\mathrm{max}}_{\mathrm{x},\mathrm{y},\mathrm{z}}}$$ 
max value of either x, y or z dimension

The minimum distance between measurement points is specified based on this parameter, determining the granularity with which the individual components are separated. If this distance is set very small, the PC will be divided into many individual components. If the *n*_pts,min_ parameter is set too low, overly fragmented components might be filtered out by the LCC algorithm and thus not considered in the CD analysis process.

The *n*_pts,min_ parameter of the LCC algorithm defines the minimum number of points required for nearby points to be grouped into the same component. If a concentration of points within a distance cell size(OL) has fewer points than *n*_pts,min_, it is removed from the dataset and no component is formed. Therefore, setting this parameter correctly is crucial for grouping measurement points to accurately represent individual (movable) objects within the study area.

The approach adopted in the investigations stipulated that objects with a volume of at least *V* = 1.0 m^3^ (assuming that objects ≥ 1.0 m^3^ can cause blockages) should be identified as components. The goal was to select *n*_pts,min_ such that the number of points roughly corresponds to the number of points in an object with a volume of at least 1.0 m^3^. Since this number significantly depends on the point density of the PC, an appropriate methodology needed to be implemented in the automated process.

The point density of the Com-PC was calculated relative to 1.0 m^3^ using density calculation in CC. The radius *r* of a sphere with a volume *V* = 1.0 m^3^ is calculated using Eq. (2).

2 $$\mathrm{r}={\left(\frac{3}{4\pi }\cdot \mathrm{V}\right)}^\frac{1}{3}={(\frac{3}{4\pi }\cdot 1{\mathrm{m}}^{3})}^\frac{1}{3}=\mathrm{0.620}\mathrm{m}$$

**Fig. 2:**
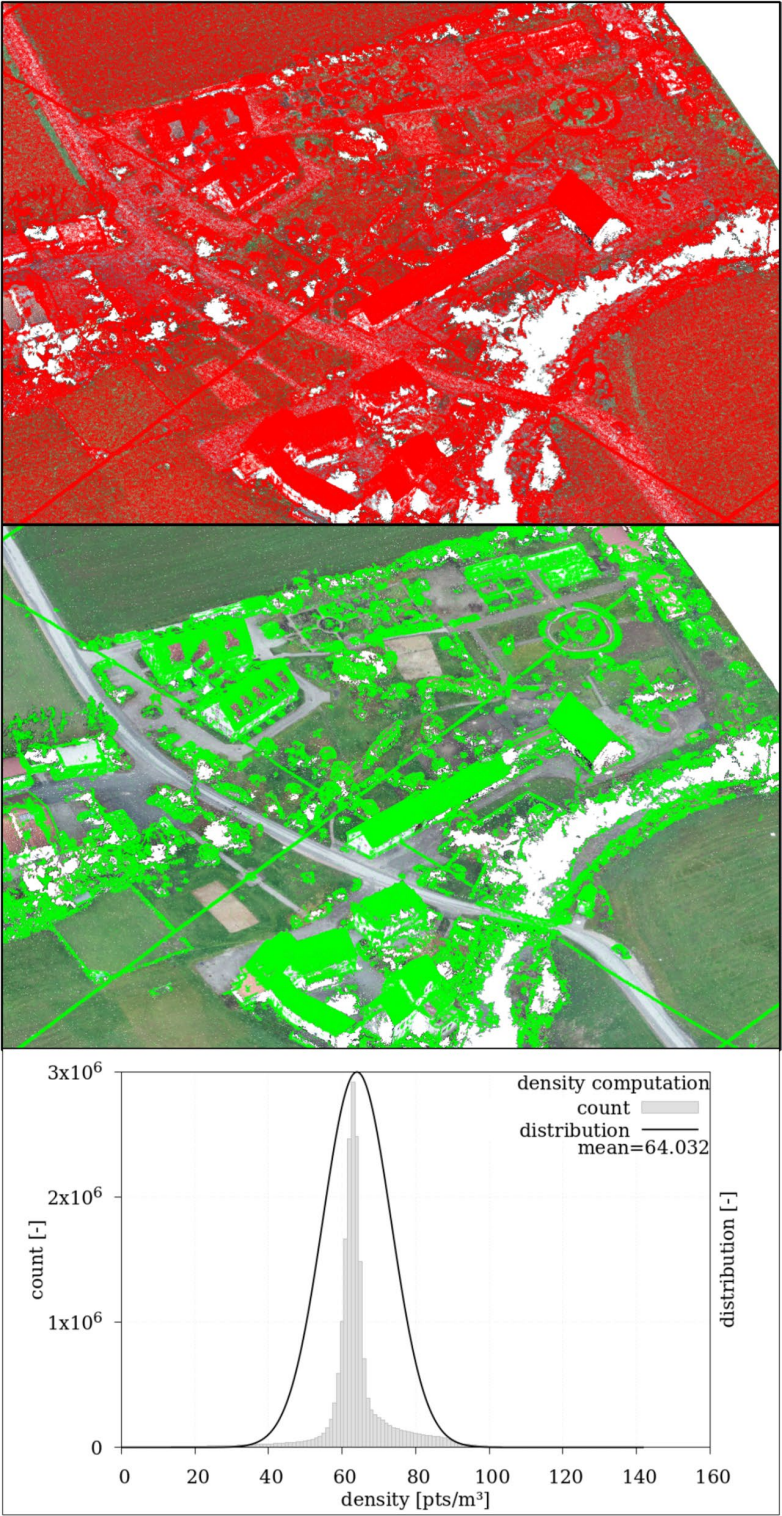
PC of the Laufer Muehle area with a normal distribution function (lower image) to determine the mean point density and marking of areas with point density ≥ 64.032 pts/m^3^ (mean point density, red marking in the upper image) and point density ≥ 66.23 pts/m^3^ (green marking in the middle image). White areas result from erroneous SfM reconstruction due to the presence of water surfaces and/or data filtering of inaccurate measurement points

To calculate the mean point density (*p*_pts,mean_) of the Com-PC, the point density for each point was determined with a radius of *r* = 0.620 m, and the average of the point density was computed. Since movable objects (usually volumetric bodies) have a notable vertical extent (*z*-coordinate), these points exhibit a higher point density than planar areas with little height variation. In Fig. 2 (middle image), areas in the study region with point density ≥ *p*_pts,mean_ (64.023 pts/m^3^ for the Com-PC in this study) are marked. Generally, these correspond to volumetric bodies and curved areas like building/roof edges. However, it was also observed that many points representing mostly flat surfaces had a point density close to the mean point density. Increasing this value to 66.32 pts/m^3^ improved segmentation of the volumetric bodies (Fig. 2, upper image).

This indicates that the *n*_pts,min_ parameter is highly dependent on the study area itself. In areas with high building density, a higher *n*_pts,min_ value is generally expected, as the point density of three-dimensional objects does not differ as significantly from the *p*_pts,mean_ In planar study areas, three-dimensional objects are easier to distinguish in the datasets.

As previously explained and illustrated in Fig. 2, this approach requires adaptation to the specific environmental conditions of the study areas. An option in the CDT module makes adapting the *n*_pts,min_ parameter possible by multiplying it with a user-specific volume factor (VF).

The *n*_pts,min_ parameter within the CDT is calculated according to Eq. (3).

3 $${\mathrm{n}}_{\mathrm{pts},\mathrm{min}}[-]=\mathrm{VF}\cdot \mathrm{V}\cdot {\mathrm{p}}_{\mathrm{pts},\mathrm{mean}}.$$

This process was implemented into the CD process through the automatic calculation of the two parameters *n*_pts,min_ and cell size (OL) and the adaptation to the characteristics of different PCs.

### Change detection tool workflow and parallel computing

For the analysis of CD, existing software solutions were used, and custom interface processes and relationships were developed and combined. Depending on the specific analysis to be performed (for example, complete CD analysis, exclusive generation of an orthophoto), adjustments to the various calculation processes can be made through parallel computing or by adjusting processor performance.

The process of the CDT can generally be divided into the following steps:

1. Generating PC for reference (Ref-PC) and compare data (Com-PC)
2. Generating orthophotos for visualization
3. Filtering data (confidence value)
4. Calculating point density for LCC analysis
5. Calculating octree level for LCC analysis
6. M3C2 distance computation, LCC analysis
7. Visualizing results (PDF)

Certain processes can be executed in parallel through the automation of processes and the capability to perform calculations on an HPC. Consequently, some postprocessing steps were divided into separate processes, even though they could have been part of a single calculation sequence (for example, confidence value filtering of the Ref-PC as part of the filtering process of the Com-PC).

**Fig. 3:**
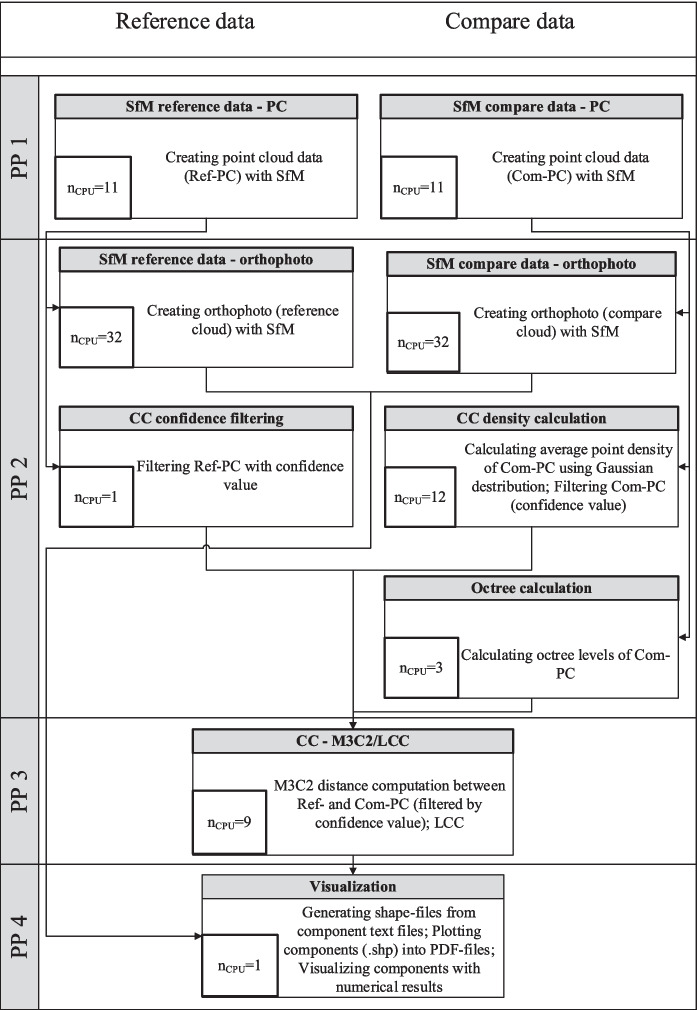
Processes of the CDT with specification of the optimized *n*_CPU_ and the parallel processes (PP)

However, there are dependencies between some processes, as certain calculation processes rely on the results of previous calculation steps. Therefore, the total computation time is significantly influenced by the maximum computation durations in the subprocesses for each parallel calculation step. The complete CDT process, the subprocesses, the dependencies between the processes, the number of used processor cores (*n*_CPU_), and the subdivision into parallel processes are depicted in Fig. 3. The number of processor cores was determined through a sensitivity analysis (the “Sensitivity analysis for optimizing the *t/n*_CPU_ ratio” section).

The implementation of all processing steps was simplified by consolidating all necessary parameters into an initialization file, which is read during each processing step. A GUI was developed using the Python library Tkinter to create the initialization file and to initialize the calculation. This allows parameter configuration and also the selection and initialization of different calculation sequences. Currently, the following CDT calculation sequences can be initialized:

1. Full CDT workflow: Complete workflow for the identification of CD based on photos from two multitemporal surveys (all processes in Fig. 3).
2. SfM reference data: SfM processing workflow for the reference data (PP1-2 reference data in Fig. 3).
3. CDT compare data: SfM processing workflow for compare data (PP1-2 compare data in Fig. 3) as well as the following postprocessing steps for CDT analysis (PP3-4 in Fig. 3).
4. SfM orthophoto compare data: Time-efficient SfM processing for the exclusive creation of an orthophoto (solely orthophoto generation of compare data in PP2 in Fig. 3).

**Fig. 4:**
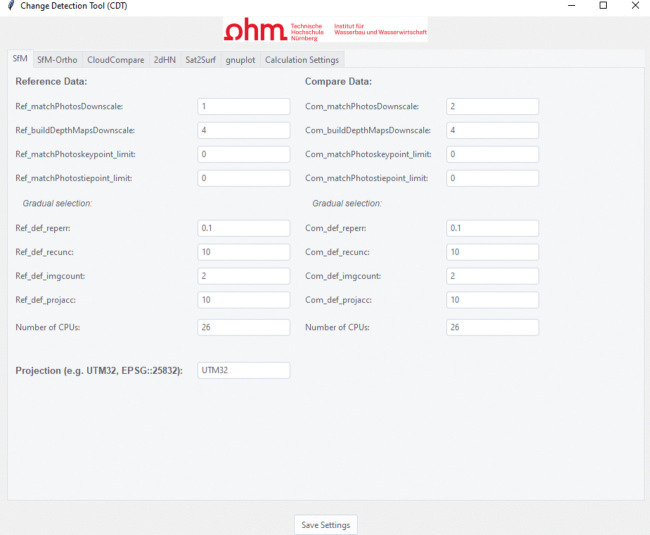
Example of the CDT input interface for the SfM calculation parameters

Additional calculation sequences can be added as needed. The parameter input is structured according to the individual processing steps, which can be accessed through the various tabs in the input interface. The parameter settings are written to the initialization file by pressing the “Save Settings” button. Figure 4 provides an example of the input interface for the SfM parameters from the CDT tool.

#### Sensitivity analysis for optimizing the *t/n*_CPU_ ratio

A sensitivity analysis was conducted to determine an optimized number of CPU per subprocess. The final visualization process for generating PDF files was excluded from this analysis, as it could only be executed on a single processor core. Calculations were performed for each subprocess with *n*_CPU_ = 1–56 (resource limit, the “Computer hardware: high performance cluster” section), and the computation time for each *n*_CPU_ (*t*) was determined (section).

#### Determining time-efficient SfM postprocessing parameters using a full factorial design experiment

A key focus of these investigations was to identify time-efficient postprocessing parameters. Studies on parameter selection for M3C2 distance determination in CC were already conducted by Kögel and Carstensen (2024). However, there are numerous parameters that significantly influence both point density, accuracy, and the necessary computation time of PCs using SfM. Thus, extensive investigations were carried out to minimize computation time while maintaining sufficient result accuracy. The ratio between the accuracy (RMS) in distance comparison between the PCs from multitemporal photos and the total duration of the calculation processes (SfM and change detection) was chosen as the indicator for this analysis.

Originally, the impact of all relevant postprocessing parameters within the CDT framework (Table 4) on the calculation outcome within plausible limits was to be examined through a Monte Carlo simulation. Effective combinations related to accuracy (ideally low RMS) and necessary computation time (ideally low *t*) were to be derived from the results. Concerning the filtering parameters of these processes (gradual selection, key and tie point variables, confidence value), it was observed that excessive filtering led to a good RMS/*t*-ratio, but the remaining points were no longer representative for the study area.

Therefore, it was decided to conduct the investigations using a design of experiments with a full factorial design, focusing on variations of the SfM parameters match photos accuracy (match photos downscale, MPD), and build depth maps accuracy (build depth maps downscale, BDD) (Table 3), which significantly influence point count, point density, and accuracy of the PC datasets.

**Table 3:** SfM accuracy/quality settings for match photos accuracy (MPD) and build depth maps downscale (BDD) processes (Agisoft LLC, 2022b)

MPD	BDD
Highest = 0	Ultra = 1
High = 1	High = 2
Medium = 2	Medium = 4
Low = 4	Low = 8
Lowest = 8	Lowest = 16

According to Antony (2010), a full factorial design experiment consists of all possible combinations of levels for all factors. As each of the two accuracy parameter (= 2 factors, MPD and BDD) could assume five different values (= levels, values in Table 3), and the investigations pertained to both reference and compare data (2 parameters per dataset); it constituted a full factorial design with five levels per variable (levels^factors^ = 5^(2+2)^ = 5^4^), totaling 625 possible parameter combinations. It was assumed that in the case of performing CDT, the processing of reference data is already completed before the acquisition of compare data as part of preparatory measures which results in *t* = 0 min for the postprocessing of reference data (workflow corresponds to the scenario and process sequence described in the “Change detection tool workflow and parallel computing” section under step 3).

The results of these investigations are presented in the “Postprocessing duration and *n*_CPU_/*t*-ration” section.

#### Combining connected components with numerical simulation results

Highly accurate georeferencing capabilities of SfM data make it possible to correlate point clouds with numerical investigation results. This was already done in Kögel et al. (2023), where three-dimensional hydrodynamic-numerical (3d-hn) calculation results were visualized using a 3d model generated from aerial imagery, which allowed for the precise determination of the influence of an FE on the adjacent terrain.

In the context of the investigations presented here, the components were combined with 2d-hn simulation results to assess the risk of mobilization during a specific FE. Existing numerical investigation results of an FE with a 100-year recurrence probability (HQ_100_) were converted into raster format, with the raster containing data points only at positions where water concentration was calculated in the numerical investigations.

It was determined whether a component was wholly or partially located within a presumed flood area using M3C2 distance comparison. If a corresponding distance of at least one measurement point of a component was within a horizontal radius of 1 m, the entire component was visualized based on the numerical results. Subsequently, a simplified assessment could be made to determine the likelihood of the onset of movement of a specific component based on the available hydraulic conditions.

The transport of solids in watercourses can generally be categorized as follows (Patt et al., 2009):

- Suspension transport
  Solid particles move within the flow without contacting the streambed. These particles settle only when the flow velocity decreases, for example, due to a sudden expansion of the cross-section.
- Bed load transport
  Bed load transport occurs near the streambed and transports solids along the streambed. This transport significantly depends on factors such as discharge, slope, bed structure, grain size, and the supply of solid material.
- Float material transport
  This refers material (mostly organic) that floats on the water surface. It often leads to the clogging of structures and hydraulic constructions and can have a significant impact on flow dynamics and water discharge.

The transport of solids in a water flow depends on a variety of factors. A key factor is the shear stress, which results from the transfer of boundary friction and resistance of a flow onto the boundary/streambed (Aigner & Carstensen, 2015; Zanke, 1982). In free flow, shear stress can be determined using Eq. (4).

4 $$\tau =\rho \cdot \mathrm{g}\cdot \mathrm{h}\cdot \mathrm{I}\cdot \left(1-\frac{\mathrm{y}}{\mathrm{h}}\right)$$

*τ*: shear stress $$\left[\mathrm{N}/{\mathrm{m}}^{2}\right]$$
*ρ*: density $$\left[\mathrm{kg}/{\mathrm{m}}^{3}\right]$$
g: gravitational acceleration $$\left[\mathrm{N}/\mathrm{kg}\right]$$
h: water depth $$\left[\mathrm{m}\right]$$
I: slope $$\left[-\right]$$
y: vertical distance to riverbed $\left[\mathrm{m}\right]$

At free surface (*y* = *h*), the shear stress has a value of zero, while at the streambed (*y* = 0 m), the shear stress reaches its maximum value. Solid transport begins when the shear stress exceeds a critical value.

The theoretical onset of movement can be determined by calculating the critical flow velocity or critical shear stress (Patt et al., 2009), while various approaches exist for determining these quantities (Aigner & Carstensen, 2015; Bollrich, 2019; Zanke, 1982).

Bollrich (2019) offers a simplified approach, presenting a correlation between various critical flow velocities and drag or shear stresses, alongside predicted material movement. Based on this approach, an estimation of potential bed load transport of movable materials in the event of an impending FE can be made through categorization of the components and comparison with numerical calculation results.

However, this approach does not consider the movement of float material. Since the onset of movement of float material can occur before the onset of bed load transport, a simplified assessment of the potential mobilization can be made based on the material categorization of individual components and the hydraulic conditions from the numerical calculation results. For example, at low flow velocities and high water levels, there is little risk of movement for stone embankments and similar building materials, whereas a pile of wood may be mobilized as float material.

#### Visualizing areas of detected changes

The conclusive visualization of changes in the study area was performed using ArcGIS Pro with the Python library arcpy and resulted in a PDF report. For each component, the visualization included the following:

- Localization within the study area
- Changes depicted using orthophotos of the study area for each of the two surveys (including marking the area of changes)
- Overlay with 2d-hn calculation results concerning expected water levels and average flow velocity

Additionally, a cover page summarized the localization of all components in the study area and the comparability of the two point clouds (reference and compared) using a Gaussian distribution of the M3C2 distance results (Fig. 11 in the “Change detection and component visualization” section).

## Results

### Postprocessing duration and *n*_CPU_/*t*-ration

**Fig. 5:**
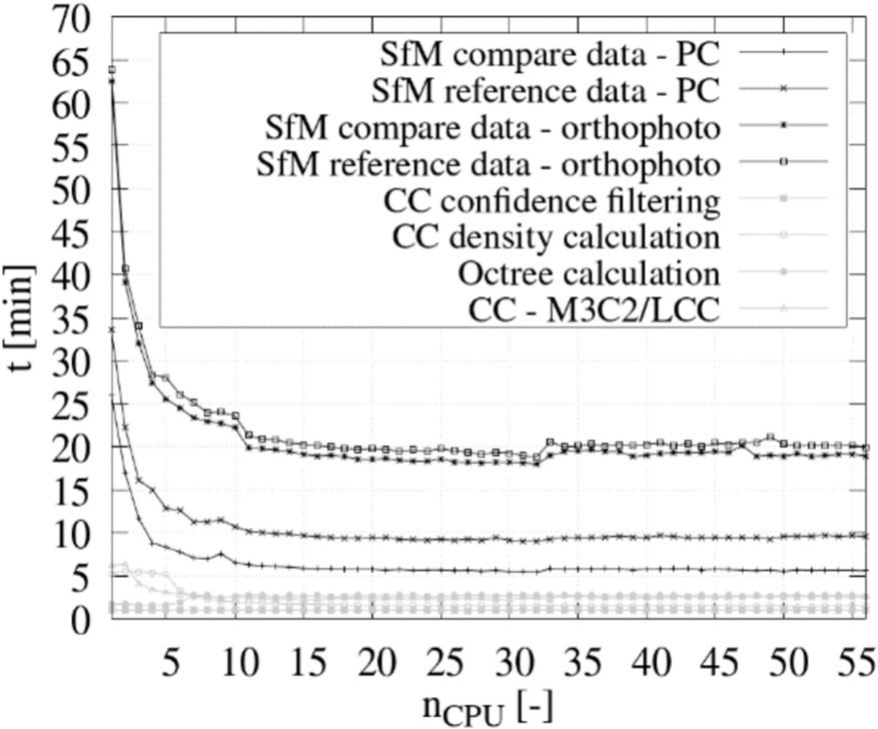
*t*/*n*_CPU_-ration for each subprocess of the CDT

The result of the sensitivity analysis to determine the *t*/*n*_CPU_ ratio is depicted in Fig. 5.

The results indicate that increasing the number of CPUs leads to a significant reduction in processing time (*t*) for all subprocesses of the CDT, particularly when *n*_CPU_ ≤ 5. For 5 < *n*_CPU_ ≤ 11, a further but less pronounced decrease in *t* is observed as the number of processor cores increases. Beyond *n*_CPU_ = 11, additional increases in *n*_CPU_ count up to 32 yield only marginal improvements in *t*. For *n*_CPU_ ≥ 33, the processing time remains nearly constant, with even higher values for *t* in the SfM orthophoto computation.

The number of CPUs used in the CC calculations has less impact on *t* compared to SfM calculations. The duration for confidence filtering remained constant regardless of the number of processor cores. For M3C2/LCC and density calculation, *t* significantly decreased with an increase of *n*_CPU_ from 1 to 9 (M3C2/LCC) or 1 to 12 (density calculation). Subsequently, the values remained approximately constant up to *n*_CPU_ = 56. For octree calculation, the best *t* values were achieved with *n*_CPU_ ≤ 6, with *n*_CPU_ = 3 yielding the best result.

The analysis provides relatively precise data for *t*, which were obtained while the HPC was not performing any other computational tasks. The actual computation time may vary depending on the overall load of the HPC, including parallel computational processes of the CDT, background processes, and other computational applications. Additionally, transferring the results to other HPC and computer systems may result in variations due to differing hardware and software configurations.

However, the analysis presented here illustrates the impact of parallel computations on the overall duration of individual processes and can be used to optimize these processes accordingly.

The CDT is intended to enable the analysis of movable objects in a study area and the implementation of appropriate measures in near real-time in the event of an imminent FE. The analysis of changes always requires a comparison with an already existing reference state, which is usually recorded before an FE. Consequently, the reference data can be processed and prepared in advance, so that some of the subprocesses shown in Fig. 3 do not have to be performed while conducting the CD analysis. This allows for a more optimized use of HPC resources and allocates more computing capacity to the remaining subprocesses.

To shorten the processing time of the CDT, this study assumes that

1. Reference point cloud and orthophoto have already been generated
2. Reference point cloud has already been filtered

The number of processor cores for each computational process were allocated based on this assumption and the previous analysis to enable parallel computation and conduct the calculation with the most effective *t*/*n*_CPU_ ratio. For most types of calculations, increasing *n*_CPU_ up to 56 would result in at least a marginal reduction in *t*. Given that the computational capacities of the HPC are finite and must be used economically, it was decided to set *n*_CPU_ to a level where no significant reductions in *t* are observed with further increases. The exception is the process of generating orthophotos of reference and compare data, as these significantly influence the overall process duration of the CDT. To reduce this duration, the capacities should be maximally utilized. Since *t* continued to decrease with increasing *n*_CPU_ up to 32, this value was chosen for the optimized implementation of the orthophoto generation process of the CDT.

**Fig. 6:**
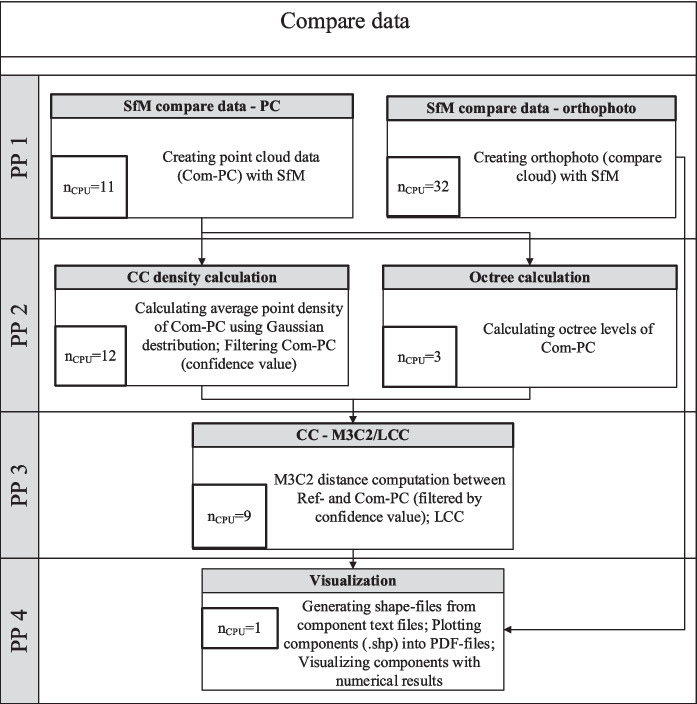
Optimized *n*_CPU_ values of the CDT and parallel processes (PP) for near real-time analysis before a possible FE

Figure 6 shows the optimized CDT workflow in the event of a potential FE (with reference data already post-processed) with visualization of the parallel processes and the optimized *n*_CPU_ determined in the “Sensitivity analysis for optimizing the *t/n*_CPU_ ratio” section.

**Fig. 7:**
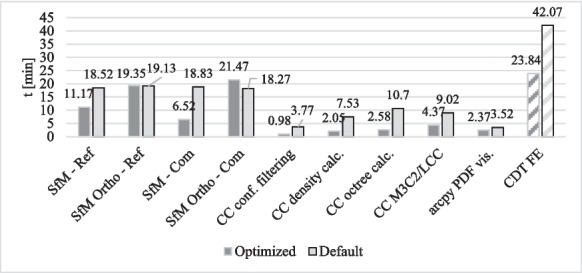
Total duration of the individual processes of the CDT (without parallel computation) and the total duration required in the event of an impending FE (sum of the corresponding subprocesses) with default and optimized values (default and optimized values each referring to postprocessing parameters and *n*_CPU_)

Figure 7 illustrates the durations of all subprocesses with an optimized *n*_CPU_ value and the resulting total duration for the optimized workflow of the CDT before a FE. For comparison, the computation durations of the default workflow with different SfM postprocessing parameters (MPD and BDD for Ref and Com datasets, each set to 2) and without the optimized use of processor cores are also depicted.

Figure 10 demonstrates that the overall durations were predominantly influenced by the orthophotos generation process, as the processing time for this task was longer than the sum of the parallel subprocesses. The high computation time for orthophoto generation is due to the fact that an additional calculation step (buildModel; generating mesh based on depth maps) is required within the SfM process, which takes up a significant amount of processing time.

In the context of the CDT analysis presented here, orthophotos serve exclusively for the photorealistic visualization of changes in the study area (Fig. 11) and do not influence the accuracy of the results or the determined components. However, the final visualization in the PDF reports can only occur after the orthophoto generation is complete, making the total process duration (for compare data) the sum of the “SfM compare data – orthophoto” and “Visualization” subprocesses. Therefore, it is essential to optimize and closely examine the SfM subprocess for orthophoto generation to further reduce the total computation time of the CDT in future studies. Since this subprocess currently serves solely for visualizing the computational results, the accuracy parameters and thus the computation times can be reduced.

Despite this, the comparison in Fig. 10 shows that the workflow depicted in Fig. 9 leads to a reduction in computation time for the optimized workflow compared to the default workflow by 43.3% (from 42.07 to 23.84 min).

**Fig. 8:**
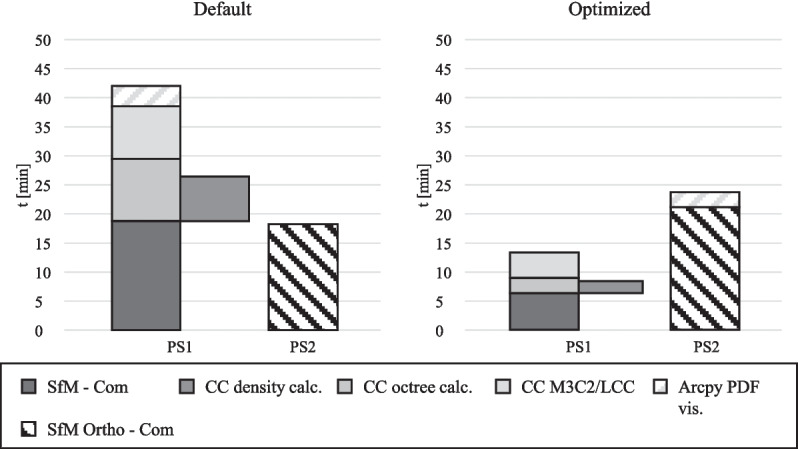
Total duration of the default and optimized workflow of the CDT before a flood event due to different dependencies of the processing sequences (PS)

The visualization process (the last processing step of the CDT) can only start once the orthophoto generation and the final process of the PC postprocessing (CC M3C2/LCC) are completed. The main difference between the total durations of the workflow with default and optimized values is that in the default process the total duration is primarily determined by the durations of the processes PC calculation, octree level and density calculation, M3C2/LCC calculation, and PDF visualization. Due to the reduced durations of these processes in the optimized workflow, the main part of the total duration results from generating the orthophotos. Since the total duration depends on the completion of both sequences, and these two processing sequences run in parallel, the overall structure of the total duration varies between the two models (Fig. 8).

If the visualization aspect based on the Com-orthophoto is omitted and the focus is solely on the identification of CD, the total duration for the optimized variant can be reduced to 15.84 min (*t* of SfM–Com + CC octree calc. + CC M3C2/LCC + arcpy PDF visualization). However, in this case, the evaluation and analysis of the components can only take place using the reference data orthophoto.

### Full factorial design experiment

The calculations based on the 625 different parameter combinations did not always yield representative results. Especially with low accuracy values, the SfM calculation was frequently terminated, resulting in no outcome for these combinations (e.g., all calculations with MPD < 2 (medium), for both Ref- and Com-PC). Overall, 181 calculation results could be derived out of the 625 parameter combinations.

The focus of the result analysis was on parameter combinations that exhibited an efficient ratio between* t* and RMS. The intersection of these combinations was chosen based on a threshold. Parameter combinations were selected that fell below both 25% of the maximum RMS value and the maximum *t* value. This reduced the number of remaining results and parameter combinations to four.

**Fig. 9:**
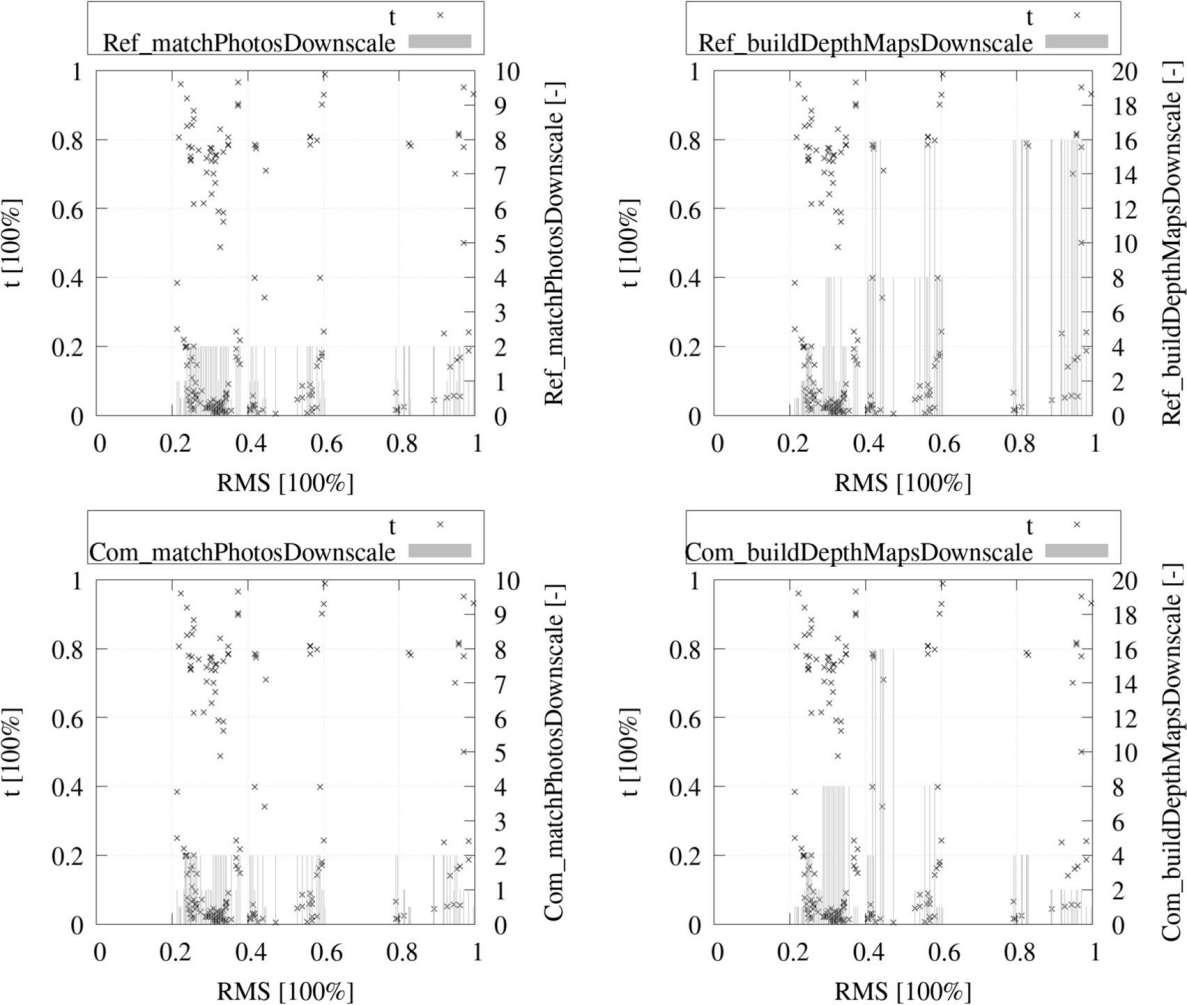
Postprocessing duration (t), accuracy parameters (match photos downscale and build depth maps downscale), and error (RMS) for reference data (upper row) and compare data (lower row) as a result of 181 different accuracy parameter combinations. The percentage values are based on the highest t and RMS values obtained from all 181 calculations

Figure 9 illustrates all results in relation to the variables MPD and BDD, with the value MPD = 0 represented as 0.5 for visualization purposes. The results show that MPD values of < 2 did not yield any results, as in these cases the SfM calculations were always aborted. Calculations could be carried out even with very low accuracy values (down to the minimum value of 16) in the case of BDD values. However, only a few results were achieved when the calculation was simultaneously conducted with BDD = 16 for survey 1 and 2. In this case, the RMS values were higher than 40%. RMS values < 25% could only be achieved with MPD ≤ 2 and BDD ≤ 4.

**Fig. 10:**
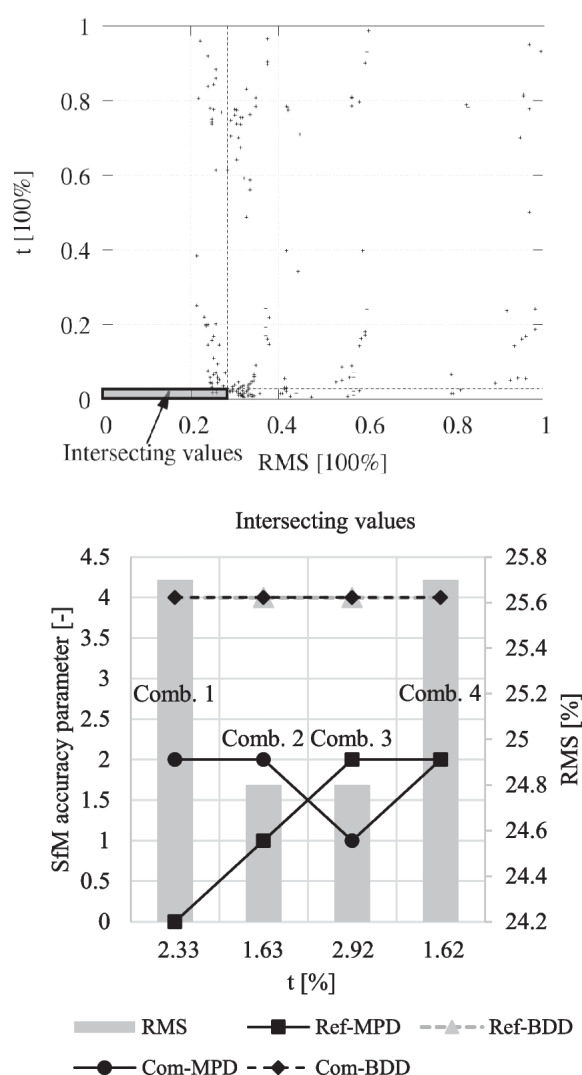
Full factorial design experiment results and value limits (25% of maximum values) with focus on the SfM accuracy values (MPD, BDD), *t*, and RMS of the four most promising results

Figure 10 depicts the results of the four parameter combinations which met the requirements. The results in both visualizations are expressed as percentage relative to the maximum values for RMS and *t* obtained from all 181 calculation results, as well as considering the workflow discussed in the “Change detection tool workflow and parallel computing” section (process sequence of step 3).

Parameter combination 2 in Fig. 10 shows the best ratio of RMS to *t*. Compared to combination 1 and 4, it has about 0.8% better accuracy, while also having a better t value compared to combination 1 and only a slightly higher *t* value (0.01%) compared to combination 4. With an almost identical RMS value compared to combination 2, the *t* value is relatively low. For this reason, parameter combination 2 was identified as the optimized parameter combination for these investigations and defined for the analysis of the presented CDT. The percentage deviations between the four final variants are relatively small, so a comparable result is also likely to be expected when considering combination 1, 3, and 4. However, since combination 2 achieved the best result, the analysis of the remaining combinations was not relevant.

The analysis in Fig. 9 show that significantly shorter calculation times can be achieved with relatively minor reductions in accuracy. The favored parameter combination was determined based on the intersection of the best RMS and *t* values (25% threshold). It is conceivable that the intersection of threshold considerations with varying percentage limits could also be evaluated. For example, accepting a higher RMS value to receive a lower *t* value might still lead to good CD results. The exact threshold value of the RMS values, beyond which an adequate representation of the connected components can no longer be achieved, was not determined in these investigations. This is because a certain RMS value must always be present due to the multitemporal surveys, and this value always depends on the area being investigated. Due to the variability of potential investigation areas, a universally valid RMS threshold cannot be determined. It is recommended that in the case of adaptation to another investigation area, an analysis under controlled conditions should be conducted and tested before analyzing CD in case of flood risk.

Table 4 presents the postprocessing parameters discussed in the “Data processing for analyzing change detection” section alongside the optimized parameters determined through the full factorial design experiment for the CDT workflow. Settings for the reference data are labeled “Ref,” while those for the compare data are labeled “Com.”".

**Table 4:** Optimized SfM processing parameters

Processing parameter	Value
SfM	
Ref_MPD [-]	1
Ref_BDD [-]	4
Com_MPD [-]	2
Com_BDD [-]	4
Match photos key point limit (Ref and Com) [-]	0 (no limit)
Match photos tie point limit (Ref and Com) [-]	0 (no limit)
Gradual selection (Ref and Com)	
Reprojection error [-]	0.1
Reconstruction uncertainty [-]	10
Number of images [-]	2
Projection accuracy [%]	10
CoudCompare	
Label connected components	
Minimum distance [m]	1.5
Object volume [m^3^]	1
Volume factor [-]	1.5
M3C2	
Projection scale [m]	0.8783
Max depth [m]	2.5
Filtering	
M3C2 distance filtering value [m]	0.25
Confidence filtering value (Ref and Com) [-]	5

### Change detection and component visualization

**Fig. 11:**
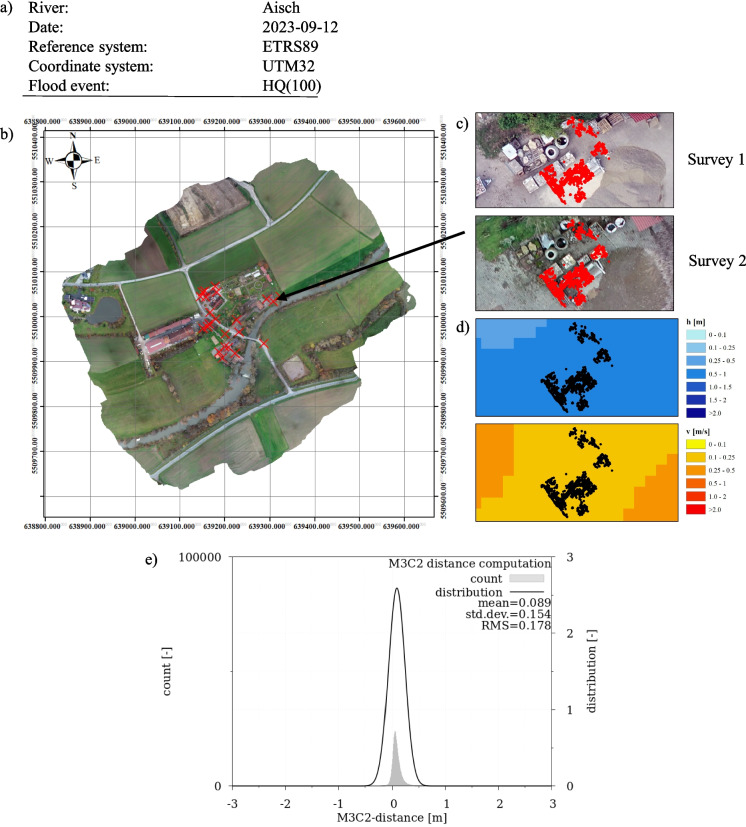
Visualization of the CDT results. **a** General information about the research area. **b** Localization of all components/areas of CD. **c** Focus on specific component (red dots) with orthophoto visualization of survey 1 and 2. **d** Combination of the component data (black dots) with 2d-hn-results (HQ_100_; water depth (h) and velocity (v)). **e** Gaussian distribution to show comparability between point cloud data of survey 1 and 2

Figure 11 depicts an edited and combined version of the report, highlighting the localization of all detected changes in the study area, along with detailed views of the components featuring RGB visualization and overlay with 2d-hn calculation results.

By segmenting into individual components and visualizing through orthophotos, it is possible to analyze changes in the study area, and to categorize and correlate them with numerical calculation results. The analysis provides an overview of potential hazards related to the initiation of movement of materials and objects in near real-time. After categorizing and integrating into the existing flow situation, it can be estimated whether movement is expected and whether appropriate measures are necessary.

A total of 23 changes/components were detected in the study area. These can be visually categorized and quantified as follows (quantity in brackets):

1. Car (10)
2. Steel container/debris pile (1)
3. Construction materials (1)
4. Other movable objects (4)
5. Faulty data/noise (7)

Since the component generation mainly focused on volume changes in the study area, motor vehicles fall into the change pattern analyzed in this CDT. Other changes detected in the critical area of an HQ_100_ event included a steel container with nearby debris pile, several blocks of stacked construction materials and other movable objects (bicycle, trash container, smaller piles). A partial displacement was recorded for the steel container, so the object was only partially categorized as movable object. Incorrect changes were detected near roof edges, where inaccurate data was produced in both surveys, leading to the identification and categorization of change.

**Fig. 12:**
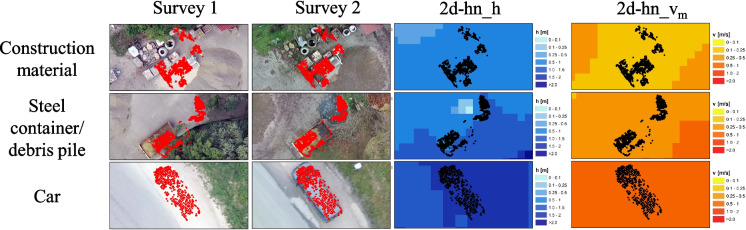
Identified components (red and black dots) in flood-prone area of a HQ_100_ flood event using the CDT and optimized postprocessing parameters. The blue images show the local water depth and the red/orange images show the mean flow velocity (results from 2d-hn-simulations)

In Fig. 12, three of the components in critical areas are visualized, along with an overlay of 2d-hn-results.

The construction materials are located in the flood area in the event of an HQ_100_, submerged in water depths of 0.5–1.0 m with depth-averaged flow velocities (*v*_*m*_) of 0.1–0.25 m/s. For the steel container/debris pile, water depths also range from 0.5–1.0 m with velocities of 0.25–0.5 m/s. The identified car was located in the area with the greatest water depth of 1.5–2.0 m and an average flow velocity of 0.5–1.0 m/s.

Estimating the begin of movement based on the assessment according to Bollrich (2019) mentioned in the “Combining connected components with numerical simulation results” section shows that the onset of material movement occurs at *v*_*m*_ ≤ 1.0 m/s for medium-sized gravelly soil and smaller grain sizes. Based on this analysis, it is not expected that the identified components will be transported during an HQ100 FE. However, this does not rule out the possibility that materials can be mobilized as floating debris.

## Discussion

### Differentiation between movable object, noise, and vegetation

Andresen and Schultz-Fellenz (2023) identify three main sources of inaccuracies in topographic point-cloud data, which must be considered and evaluated in relation to change detection: positional accuracies (e.g., sensor precision, registration), point-cloud classification (e.g., bare-earth extraction), and surface representation (e.g., resolution, interpolation). Considering these three factors, accuracy must be evaluated in relation to change detection. They also state that determining accuracy in the context of a multi-temporal change detection analysis is challenging due to the varying conditions of the study area, as deploying GCPs, which are typically used to assess data accuracy, is a time-intensive and logistically challenging task. This is especially difficult in the case of an FE, where terrain accessibility is limited, and staying in the flood zone may be life-threatening.

However, they also explain that recent advances in RTK positioning have mitigated the need for GCPs within certain accuracy ranges. In the studies presented here, multi-temporal data were aligned based on the RTK positioning of the measurement device. The accuracy of the study data was demonstrated in Kögel and Carstensen (2024) through comparison with GCPs, with deviations within the SAPOS (2015) specifications (1–2 cm horizontal accuracy, 2–3 cm vertical accuracy).

In the case of fixed monitoring systems, such as those described by Blanch et al. (2020) and further developed in Blanch et al. (2021, 2023), positional inaccuracies can be largely minimized. They illustrate how close-range photogrammetry data from fixed camera systems, combined with post-processing algorithms and validated using terrestrial laser scanning data, can achieve change detection accuracy within just a few centimeters. However, applying these algorithms to UAV data proves challenging, as capturing images from exactly the same position with high temporal frequency is not feasible.

The surface representation depends on the resolution of the datasets, which is significantly influenced by the measurement system and the distance to the object of investigation. Point distances in the range of a few millimeters can be achieved with LiDAR systems, enabling highly accurate change detection analyses (Kromer et al., 2017; Winiwarter et al., 2020). However, Baltsavias (1999) describes that, due to the laser footprint, it is impossible to image a whole area homogeneously and without gaps and overlaps.

In camera-based measurement systems, the sensor resolution (number of pixels), focal length, and the distance to the object of investigation are the parameters that determine the ground sampling distance (GSD), which is used as a measure of imaging accuracy (Karamuz et al., 2020). In this study, the GSD was 3.01 cm/pixel.

Although wide areas can be captured with satellite data, the resolution of modern satellite systems is in the range of ≥ 1 m (Colacicco et al., 2024; Kuntla, 2021). Consequently, these systems are primarily suited for large-scale representations, such as surface water mapping (Markert et al., 2018; Pham-Duc et al., 2017; Shen et al., 2022), flood mapping (Sipelgas et al., 2021), or the generation of digital elevation models (Braun, 2021).

However, the detection of smaller, dynamic objects, typically with diameters or circumferences of only a few meters, remains highly limited with these systems.

The CDT presented in this study is capable of identifying changes with high accuracy and designed to detect differences while accounting for seasonal effects and varying vegetation conditions.

The extraction of the bare-earth surface is a significant challenge in the field of remote sensing. Dyer et al. (2020) describe vegetation obstructions of the surface as one of the primary disadvantages of using UAVs for mapping river features. Both seasonal growth and the movement of vegetation can lead to erroneous results in change detection and must be considered in the change detection process.

The identification of vegetation is highly dependent on the accuracy of the measurement data. Even with high data precision and good alignment between the multitemporal datasets, minor movements of the vegetation can lead to significant changes. To neutralize discrepancies resulting from the aforementioned inaccuracies, changes of ± 0.25 m were filtered in this study, with differences > 0.25 m classified as significant changes. The CDT is capable of identifying moving objects with an accuracy range from less than a decimeter (considering GSD = 3.01 cm/pixel and horizontal/vertical accuracy of 1–3 cm) to large-scale objects spanning several meters. This enables the tool’s primary application: identifying objects that could cause blockages and damages to infrastructure (Martín-Vide et al., 2023) and pose risks to human life during a FE. The use of UAVs, in comparison to the aforementioned remote sensing systems, offers a well-balanced compromise between accuracy, flexibility, and coverage area. This allows for effective risk assessments with short lead times before a flood event.

### Combination of 3d change detection and visual analysis

The analysis using the CDT is based on a combination of 3d difference determination and visual assessment through orthophotos. This approach requires minimal preprocessing and allows the tool to be applied across a wide range of study areas. In contrast to AI-based semantic segmentation via machine and deep learning, which requires a large dataset to train algorithms and involves a time-consuming training process, this method offers significantly more flexibility. For instance, Farmakis et al. (2022) employed a deep neural network with a 5-year change detection database consisting of over 8000 rock slope clusters to identify changes during the training phase. Similarly, Gebrehiwot et al. (2019) note that “training a deep CNN from scratch with a small dataset is not always advisable due to poor classification results and overfitting.” They further report that their study achieved good classification results “even though only a hundred UAV images were available for training.” This highlights that AI-based analysis methods require a very high data density to achieve sufficiently accurate results across different research scenarios. AI-based semantic segmentation is primarily applied to 2d datasets, limiting the ability to capture the three-dimensional characteristics of moving objects and accurately represent their true dimensions.

The categorization of changes detected by the CDT is conducted through the visual analysis of orthophotos from two multitemporal surveys. By performing an orthogonal comparison of these images, moving objects can be effectively identified and classified. However, the accuracy of object classification depends on the subjective assessment of the operator, which may lead to variations in conclusions depending on the evaluator.

### Field of application

The application of the CDT through UAV-based surveying offers the advantage of flexible deployment across various study areas. Particularly when combined with SfM, it enables surveying with high spatio-temporal resolution. While the high resolution of LiDAR data generally allows for precise three-dimensional measurements, UAV- or airborne LiDAR systems cannot derive RGB color information from the data. As a result, this system can only categorize changes based on geometric attributes, making object definition challenging.

UAV-based SfM remote sensing facilitates the analysis of movable objects and the implementation of corresponding measures before and during a FE to mitigate damage. This capability allows for a rapid and spontaneous response to the constantly changing conditions that occur during a FE.

In contrast to stationary measurement systems, such as described by Blanch et al. (2020, 2021, 2023) or Gabrieli et al. (2016), UAV-based systems offer high flexibility, allowing for precise evaluation of designated areas within a river basin. However, since FEs are often accompanied by heavy rainfall and extreme weather conditions (such as rain and wind), the operational capability of a UAV during a flood event is not always guaranteed. Generally, the CDT is intended for application prior to a potential flood event, thereby minimizing the likelihood of encountering extreme weather conditions during its primary use. However, it is advisable to employ a measurement system in remote sensing related to impending or ongoing FEs that can operate without damage under adverse weather conditions.

Modern satellite systems can capture vast areas spanning several square kilometers in a single image, enabling extensive coverage among remote sensing units, while SAR satellite systems can collect data independently of weather conditions and time of day (Shen et al., 2022).The accessibility of satellite data depends on the satellites’ orbits and acquisition intervals, which significantly limits the frequency of data collection. Consequently, it cannot be guaranteed that a relevant satellite image of the study area for change detection analysis will be available prior to a FE. The challenges associated with imaging in urban areas are discussed by Refice et al. (2022). They explain that SAR operates as a side-looking instrument, which may result in substantial areas of urban terrain being obscured due to radar layover and shadows created by buildings. In contrast, the close proximity of UAVs to the ground, along with their ability to capture images from various perspectives and angles, reduces shadowing effects and facilitates the detection of changes even beneath canopies.

**Fig. 13:**
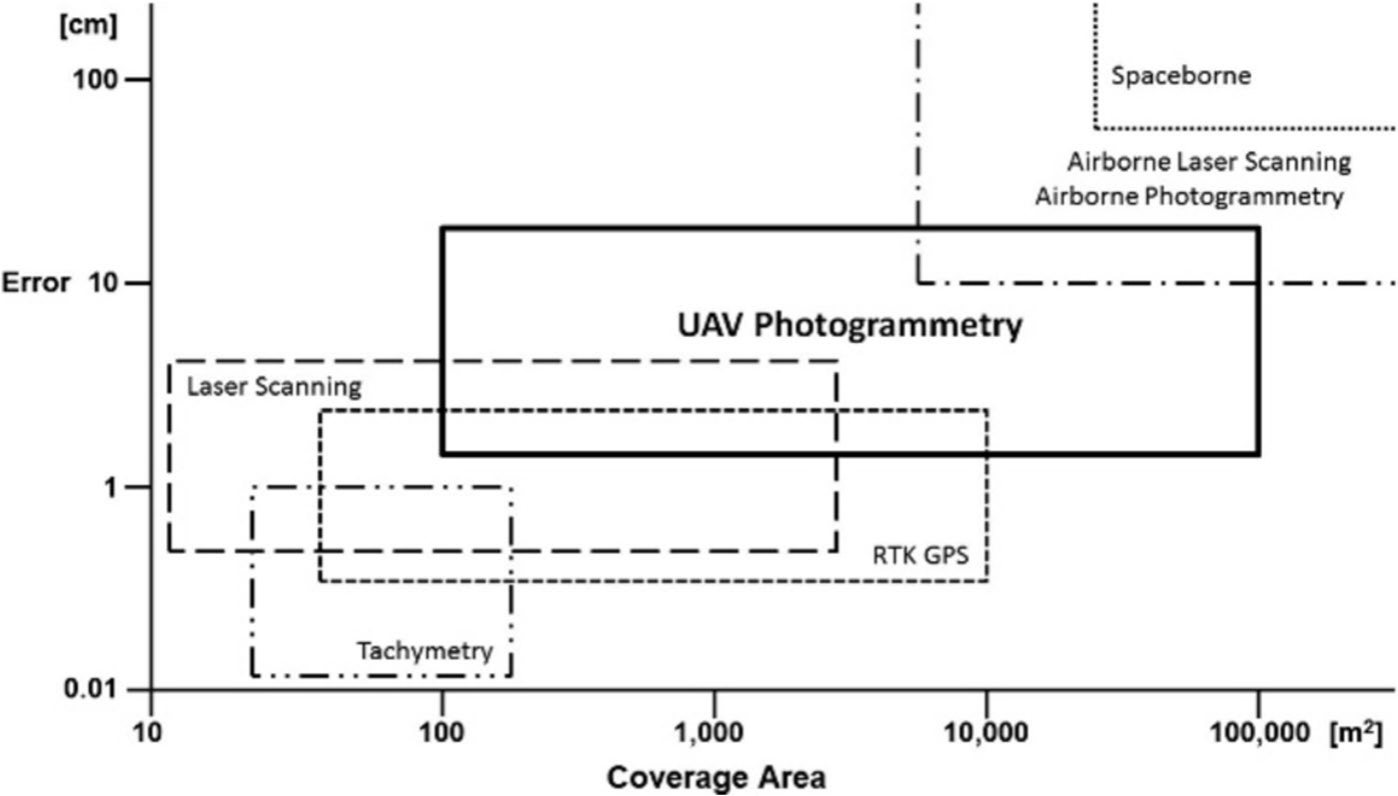
UAV application possibilities in surveying tasks (Siebert & Teizer, 2014)

UAV photogrammetry represents a methodological middle ground in terms of accuracy and coverage area, situated between high-precision measurement techniques such as tachymetry and terrestrial laser scanning, and broader measurement systems like spaceborne or satellite systems (Fig. 13). It can be concluded that, despite UAV photogrammetry exhibiting lower accuracy compared to other measurement methodologies, it is still sufficient for identifying potential blockage objects.

### 2d-hn-based risk assessment

The analysis of material transport, whether in the context of suspension, bed load, or float material transport, is a complex task that cannot be universally conducted. Due to three-dimensional hydrodynamic (turbulence) effects, as are practically always present in natural watercourses, focused investigations are required for precise statements. The estimation of the initiation of movement for a detected change using the CDT relies on a comparison with the flooded area from an existing 2d-hn simulation of a FE with a specified return interval HQ_T_. Typically, 2d-hn simulations provide insights into parameters such as flow velocity, water depth, and flow direction. By comparing these findings with established approaches (Bollrich, 2019), a simplified estimation of the onset of material movement can be made.

However, concerning the initiation of movement induced by flooding, there are approaches that demonstrate the complexity of field conditions. Keller and Mitsch (1993) developed a theoretical model to determine the point at which a submerged vehicle becomes unstable by analyzing the various forces acting upon it, including the friction coefficient, fluid density, drag coefficient, buoyancy force, and the projected area of the vehicle facing the flow. Notably, their findings indicate that the physical relationships cannot be universally defined within this mathematical framework.

Gou et al. (2022) analyzed the instability of specific car models in floodwaters using a probabilistic approach and validated their findings with five experimental datasets. These studies established the relationship between critical flow velocities (drag force) and water depths (buoyancy force) beyond which sliding movement occurs. These theoretical models offer a more complex method for assessing the initiation of movement in vehicles due to flooding. However, the results derived from studies conducted under ideal conditions (characterized by low turbulence and precise information regarding flow direction, velocity, and water depth) can only be partially applied to the turbulent and dynamic characteristics of actual flood events.

The studies indicate that a comprehensive understanding of various properties of moving objects is essential for accurately assessing the onset of movement induced by flooding. In the context of a FE and the change detection analysis conducted in this study, it is possible to categorize the identified changes broadly; however, this categorization does not directly provide information about the physical properties of those objects. Given that timely assessments are crucial for risk evaluation, complex analyses, such as those described by Keller and Mitsch (1993), cannot be undertaken in the immediate lead-up to a flood event.

The CDT is capable of identifying moving objects and establishing a connection between their location and predicted flow parameters. Particularly through the correlation with water levels and the assessment of whether the detected moving object is floating material, reliable statements regarding flood risk assessment can be made.

Furthermore, the integration into hydrodynamic models was based on existing numerical investigation results and considering a predefined FE (HQ_100_) and thus dependent on prediction models. Consequently, modeling inaccuracies (both in prediction and numerics) may lead to further discrepancies from natural conditions.

Regardless of this fact, statements about the presence of movable objects in the influence area of an FE can be made in a short time, and corresponding (precautionary) measures can be taken, thereby potentially reducing damage and hazard potentials significantly.

### Near real-time risk assessment

Flood risk assessments are typically based on flood risk and hazard maps (LAWA, 2010, 2018). These maps illustrate the extent of inundation areas and the infrastructure affected during specific flood events, serving as the foundation for flood risk management plans (LAWA, 2019). The necessity of removing exposed risk elements from hazardous zones has been postulated by Müller (2010). The CDT using UAV-SfM is capable of identifying such elements shortly before a flood event and representing their exposure to various flooding scenarios. Due to the short analysis duration (data collection and evaluation < 1 h for an area of 0.4 m^2^), the tool can respond effectively to short-term weather forecasts.

Currently, the analysis requires prior data acquisition (reference data) to enable comparison between the two states. Consequently, the areas to be monitored must be defined well in advance of a flood event, resulting in a loss of flexibility. In this context, it is essential to explore the application of the CDT using publicly available datasets as reference data. The increasing availability of high-resolution terrain data presents an opportunity to obtain reference data without the prior specification of a study area. However, it remains to be evaluated to what extent these datasets are comparable to SfM data and the accuracy with which changes can be detected.

The risk assessment of detected changes is heavily dependent on the quality of flood forecasts and the two-dimensional hydrodynamic analyses. The greater the time interval between the forecast and the predicted flood event, the less precise the assessment becomes. For instance, 3 days prior to the event, the accuracy is approximately 75%, while it increases to about 90% when assessed 24 h before the event (Müller, 2010).

The true extent of a FE can only be determined with sufficient probability over time. The results obtained using CDT should take this into account, and, if necessary, the localization of detected changes should be evaluated based on flood areas from FEs of varying intensities.

Assuming a flight duration of approximately 15 min (Kögel & Carstensen, 2024) and a turnover time of about 5 min (return flight, reading data from memory card, copying data, battery swap), the UAV can survey an area of 0.4 km^2^ every 20 min and, by postprocessing with the CDT, produce a change detection result every 44 min (the “Postprocessing duration and *n*_CPU_/*t*-ration” section).

Thus, a change detection analysis is also feasible immediately before an FE and can be adjusted to the flood conditions, depending on forecast models.

## Conclusions and future prospects

By optimizing SfM postprocessing, a parameter combination was identified that significantly accelerated the detection of changes. In comparison to standard postprocessing parameters, the processing time was reduced by 43%, from 42 to 24 min. Considering a survey duration of approximately 15 min (survey parameters according to Kögel and Carstensen (2024)), along with an estimated changeover time of around 5 min (for drone return flight, battery replacement, and data card retrieval), change detection can be completed in a 0.4 km^2^ area in approximately 44 min.

Compared to other remote sensing systems, UAV-SfM has been found to effectively integrate accuracy, coverage area, and postprocessing efficiency, making it a valuable method for conducting change detection analyses in the context of flood risk assessment prior to an FE. Utilizing process optimization and the visualization capabilities of CDT allows for the estimation of the onset of movement of movable objects at high temporal resolutions through simplified analytical approaches.

Further improvements can be achieved in both coverage area (using fixed wing UAVs, Dyer et al. (2020)) and postprocessing duration. Currently, the generation of orthophotos constitutes the majority of the CDT's postprocessing duration, despite being used solely for the visualization of detected changes. Optimizing this process has the potential to reduce the overall postprocessing time significantly.

The implementation of the CDT is viable for a wide array of UAVs equipped with RGB camera systems, requiring minimal preparation. By combining 3d change detection with RGB visualization, this tool can be applied across various study areas. However, the tool does not facilitate automated categorization of changes, resulting in risk assessment being subject to the operator’s subjective judgment.

During the investigations, different components were identified in areas that could be influenced by an HQ_100_ event, while three of these components (car, construction material, steel container/debris pile) were located in particularly heavily flooded areas. Based on the categorization and hydraulic conditions (water level and mean flow velocity), it could be estimated that there is likely no risk of mobilization. However, this estimation currently relies on a simplistic approach regarding the onset of sediment transport. Research has demonstrated that assessing the initiation of movement triggered by flood impacts requires a complex three-dimensional analysis that accounts for a multitude of parameters. Within the context of flood preparedness, conducting such a comprehensive evaluation is often impractical due to the limited lead time associated with flood events. Moreover, the varying characteristics of different movable objects complicate the ability to make universally applicable estimations regarding the actual onset of movement. Consequently, while a simplified estimation may be practical, it is essential that future approaches also incorporate considerations of the floating behavior of objects.

Optimizations of change detection accuracy can be achieved through co-registration. Possible errors and difficulties in this regard have already been addressed in the “Point cloud postprocessing” section. However, through preparation and adaptation to the conditions of a study area, corresponding optimizations can be made. For example, the point clouds from the surveys to be compared can be filtered based on unique and consistent reference points (such as infrastructure/buildings), and the transformation matrix of the two filtered point clouds can be determined through co-registration of these reference points. This would ensure that the co-registration process is not conducted based on changes in the study area, thus preventing a faulty result from being generated. The determined transformation matrix could be applied to the unfiltered point clouds, leading to a better overlap of the PCs of multitemporal surveys. This approach has not been incorporated into the existing workflow of the CDT, as the analyses presented in this study aimed to minimize preparation efforts and ensure applicability across a diverse range of study areas. However, in future investigations, the implementation of this process should be explored.

The CDT has not yet been applied to different regions. While the use of an RGB UAV system with RTK connectivity generally allows for the transfer of the CDT to various locations, its applicability to areas with differing structural characteristics (e.g., densely or sparsely populated regions, or those with more complex infrastructure) has not yet been examined. This will be the focus of future investigations.

The analysis and detection of the water surface within the framework of the CDT should be enabled and improved in future investigations. Currently, an orthophoto of the study area is created to visualize the water surface, but no further measurements have been made on this basis. Additionally, no analyses of the accuracy of the orthophotos or optimization possibilities in postprocessing have been conducted in this regard. However, it is evident that UAVs are suitable for “[…] gathering information on flooding conditions and dynamics and determining the extent of floods […]” and “[…] increasingly used in geohazard studies and monitoring” (Vélez-Nicolás et al., 2021). High-frequency near real-time analysis of flood extent would enhance the capabilities of the CDT, making it an even more valuable tool for flood risk management by enabling the rapid implementation of measures and the subsequent assessment of a flood event.

## Data Availability

The datasets generated during and/or analyzed during the current study are available from the corresponding author upon reasonable request.

## Code availability

After the CDT has been supplemented with additional features and optimized, it is planned to make the code available upon request from the corresponding author.
